# An Ecological Monitoring and Management App (EMMA) for Older Adults With Chronic Pain: Protocol for a Design and Feasibility Study

**DOI:** 10.2196/26930

**Published:** 2021-08-26

**Authors:** Katharina Ledermann, Omar Abou Khaled, Maurizio Caon, Thomas Berger, Joelle N Chabwine, Joachim Wicht, Chantal Martin-Soelch

**Affiliations:** 1 Department of Consiliar and Liaison Psychiary University Hospital Zurich Zurich Switzerland; 2 Department of Psychology University of Fribourg Fribourg Switzerland; 3 Human Tech Institute School of Engineering University of Applied Sciences and Arts Western Switzerland Fribourg Switzerland; 4 Digital Business Center School of Management University of Applied Sciences and Arts Western Switzerland Fribourg Switzerland; 5 Department of Clinical Psychology and Psychotherapy University of Berne Berne Switzerland; 6 Division of Neurorehabilitation Fribourg Hospital Fribourg Switzerland; 7 Neurology Unit Department of Neuroscience and Movement Science University of Fribourg Fribourg Switzerland

**Keywords:** chronic pain, older adults, mHealth, online intervention, self-management

## Abstract

**Background:**

Chronic pain is a complex problem for many older adults that affects both physical functioning and psychological well-being. Mobile health (mHealth) technologies have shown promise in supporting older persons in managing chronic conditions. Cognitive behavior therapy is recommended for older people with chronic pain. However, web-based treatment programs for chronic pain are not aimed at the needs of older people and offer standard therapies without providing tailored treatment for this population.

**Objective:**

To address this problem, we aim to develop a psychological web-based intervention for ecological monitoring of daily life experiences with chronic pain called EMMA to support self-management of chronic pain in older adults.

**Methods:**

The key clinical and engagement features of the intervention were established through the integration of evidence-based material from cognitive behavioral therapy for the treatment of chronic pain in older adults. The development process uses a co-design approach and actively involves end-users in the design process by incorporating feedback from focus groups with older adults in order to inform a user-centered intervention design. For the co-design process, we will include 10 older adults with chronic pain, who will discuss the requirements for the app in workshops in order to ensure suitability of the app for older adults with chronic pain. In order to test the feasibility and acceptability of the intervention, we will include a sample of 30 older adults with chronic pain who will test all features of the intervention for a period of 8 consecutive weeks. After the trial period, validated instruments will be used to assess usability and acceptability, as well as influence on pain levels and associated physical and psychological symptoms. Participants will be invited to take part in a semistructured telephone interviews after the trial period to explore their experiences using the app.

**Results:**

Digitalization of the pain diary and psychotherapeutic content has started. Recruitment of participants for the co-design workshops will start as soon as we have a functioning prototype of the electronic pain diary and EMMA intervention, which is expected to be in September 2021. The feasibility study will start as soon as the co-design process is finished and required changes have been implemented into the pain diary and the EMMA intervention. We expect to start the feasibility study early in 2022.

**Conclusions:**

Required changes to assure usability and acceptability will be directly implemented in the app. EMMA brings together a strong body of evidence using cognitive behavioral and self-management theory with contemporary mHealth principles, allowing for a cost-effective intervention that can be used to target chronic pain anywhere and anytime by older adults. Given the ubiquity of mHealth interventions for chronic conditions, the results of this study may serve to inform the development of tailored self-management interventions.

**International Registered Report Identifier (IRRID):**

PRR1-10.2196/26930

## Introduction

This paper presents a new psychological web-based intervention for chronic pain in older adults in the form of a web-based self-guided intervention called EMMA (Ecological Monitoring and Management App) to support self-management of chronic pain.

Chronic pain is one of the most common conditions affecting older adults over the age of 65 [1]. Previous studies [2] on pain in older adults have reported prevalence rates from 24% to 72% and a high prevalence among residents of nursing facilities. Older adults with chronic pain are likely to experience more physical impairments and interference with activities than young people, and pain locations tend to spread over the whole body with age [3]. Commonly occurring pain disorders that affect older adults include back pain, osteoarthritis, rheumatoid arthritis, musculoskeletal pain, and various neuropathies from diabetes, herpes zoster, chemotherapy, or surgery [3]. Pain is also common in the advanced stages of many chronic diseases, such as congestive heart failure, end stage renal disease, and chronic obstructive pulmonary disease [3]. Furthermore, many joint repair and replacement surgeries are performed annually, and a percentage of patients undergoing these procedures report chronic pain despite surgery [4].

Unrelieved pain in older adults has been shown to cause or increase depression, anxiety, and sleep disturbance; decrease mobility; and increase social isolation [5], as well as being a significant risk factor for decreased functional capacity and onset and progression of disability in old age [3]. The global burden of chronic pain is projected to increase as prevalence rates for pain increase as the global population continues to age. Older patients are the most likely of all age groups to receive inadequate pain treatment [6]. Internationally, there has been increasing recognition of the importance of developing nonpharmacological interventions for older adults with chronic pain [7].

Current best practices for the management of chronic pain in older adults involves multidisciplinary cognitive behavioral interventions that focus on fostering self-management [8]. Studies in older adult chronic pain have shown that comprehensive interactive face-to-face psychoeducational interventions lead to symptom reduction and improved health-related quality of life compared with care that is strictly medication focused, with a postulated mechanism being enhanced self-efficacy and empowerment over disease and symptom management [9]. Self-care strategies for managing stress, facilitating positive coping behaviors, or reducing anxiety may be especially effective for mediating psychological and social components of pain management; evidence supporting self-management treatments in improving pain and pain-related disability in older individuals with chronic pain is strong [10]. However, cognitive behavior therapy techniques are underutilized, particularly among older adults with chronic pain [11]. Access to effective pain management programs is often limited, due to lack of services, lack of patients’ resources, and fear of stigmatization [11]. Other barriers to treatment include physical symptoms that limit mobility, clinic distance, transportation requirements, and cost constraints. Self-management interventions, such as web-based interventions, are a promising alternative to traditional treatments and have the potential to overcome barriers and improve the availability of effective psychological treatments.

Several studies [12] have piloted web-based self-care interventions for chronic conditions in older adults such as diabetes, dementia, and osteoarthritis [12]. Results from these studies suggest that the benefits of these web-based self-care interventions appear to be comparable to in-person interventions [12,13]. There is a growing number of mobile pain management apps available in app stores such as the Apple App store and the Google play store. Several systematic reviews confirmed a beneficial effect of these apps for pain management in terms of reduced pain intensity and increased quality of life [14,15]. However, most apps did not use evidence-based strategies and were developed without the involvement of health care practitioners, and none included end-users in the development process [14]. Furthermore, to the best of our knowledge, no web-based self-management interventions with a specific focus on chronic pain in older adults exist. A recent review [16] on the quality and usability of arthritic pain self-management apps for older adults concluded that only a few of the currently available pain apps offer a comprehensive pain self-management approach incorporating evidence-based strategies in accordance with published guidelines and take into account the needs of older adults in terms of usability.

The aim of this paper is to describe the development of a theoretically sound psychological web-based self-management intervention based on ecological monitoring of daily life experiences. The EMMA intervention will address all etiologies of chronic pain in older adults. The EMMA intervention will be adapted to the needs and challenges of older adults with respect to psychological content, as well as web design. The developmental process will follow methods in accordance with the Medical Research Council framework for development and evaluation of complex interventions [17].

## Methods

### Overview

The study will be subdivided into 2 phases, namely, (1) the development of the clinical content of the web-based intervention, including the digitalization process, and (2) the feasibility study.

The goal of EMMA is to provide automated support, in terms of self-management for chronic pain in older adults, that has been carefully designed alongside members of the target population, as these are key drivers of app usage within the Ritterband model [18], the state-of-the-art model for the development of web-based interventions. We defined a clear set of requirements for the development of the app based on literature reviews [19]: The app must be capable of (1) meeting the needs of older adults in terms of presentation, user-friendliness, acceptance, and user guidance by using a user-centered design and actively involving older persons in the development process, (2) supporting both major mobile operating systems (Apple iOS and Google Android), (3) allowing clinicians to access the pain data in a graphical format for patient care in clinic using either the app or the web, and must be (4) compliant with data protection laws in Switzerland, including the Swiss Federal Data Protection Law [20] for the privacy and security of protected health information, as well as European Union Medical Device Regulation [21] and (5) usable as a stand-alone treatment or incorporated in a multidisciplinary pain management practice. EMMA provides psychoeducational material and comprehensive modules to teach self-management strategies for chronic pain in the older adults to enhance self-efficacy, a primary determinant of behavior change [22].

### Development Aims

#### Features

Currently, there are no widely accepted frameworks or clinical guidelines recommending the key components of a pain self-management program in general or for older adults specifically [23]. Based on a systematic review [16] of self-management programs on chronic pain in older adults, previous web-based interventions for chronic pain [15], and our own clinical practice, we selected tools that were known to be effective, relevant, and usable. The clinical content of EMMA, is grounded in cognitive behavioral therapy [24], the biopsychosocial model of chronic pain [25], and self-management theory [26]. Furthermore, we put an emphasis on experiential learning, given that older adults have been shown to learn more from active training than from didactic content [24]. Although the number of randomized controlled trials investigating mind-body interventions (mindfulness-based stress reduction, yoga) for older adults with persistent pain is small [27], research suggests that these interventions are effective for enhancing well-being, mood, and pain acceptance [27]. Therefore, we aimed to incorporate mind-body techniques such as breathing techniques, mindfulness and gratitude practices, and yoga for older adults in our program.

The EMMA intervention will contain psychoeducational material in video format, a digital pain diary, and self-management treatment modules.

Psychoeducation is an essential part of pain management for older people [6]. Therefore, we will include psychoeducational information in short, easy-to-understand explanatory videos with a maximum duration of 120 seconds. The videos will be high contrast, limited to the most important information, and provided with subtitles to be accessible to older adults with hearing and visual loss. The videos will be embedded in the app and will contain information about the development of chronic pain, the pain-self-management process, medication use, communication with health professionals, adaptive coping strategies, fatigue, sleep, fear of falling, importance of activity, emotion regulation, attitudes and beliefs about pain, psychological issues management, and emotion regulation. There will also be a section on the influence of pain on the family, partners, or others. The video material will be based on validated psychoeducational pain management courses [28] and adapted to address the specific needs of older adults. The content will be digitalized with the help of research assistants in Psychology at the University of Fribourg.

The digital pain diary is based on a paper-and-pencil diary that was developed in a pilot study on the experience of chronic pain in daily life at the University of Fribourg and tested in a sample of 60 patients with chronic pain and 40 healthy individuals [29]. The diary covers different aspects of the painful sensation (sensory, affective, cognitive and behavioral). The following data will be collected by the pain diary: (1) pain intensity, (2) quality of painful sensation, (3) emotional state, (4) thoughts, (5) stress, and (6) activity. The content of the diary will be digitized as part of the project and adapted to provide an original set of questions that we know will be suitable for repeated questioning during everyday life for ecological momentary sampling. The content of the paper-and-pencil diary has been found suitable for repeated questioning during everyday life for ecological momentary sampling; therefore, it will be converted to a digital form as part of the project. Because regular monitoring of pain levels and associated symptoms and experiences is the foundation upon which other changes in behavior are built, reminders will be added to the app, that prompt users to log their pain 4 times per day (by default: after waking up, noon, evening, before going to bed) [30]. Pain logs for self-monitoring are a useful source of clinically relevant information about pain intensity and quality; key activities; emotional state, stress, and thoughts; and the relationship between them and the context in which they occur. This method allows the measurement of pain and related factors in real-world settings, which possibly enhances accuracy and detail of measurement and helps to identify potential mediators and moderators of pain in older populations. Prior studies [31] have found strong support for the feasibility and application of this method in healthy older adults, people with clinical disorders, and patients with chronic pain. Furthermore, studies emphasize the importance of self-observation methods for understanding the different factors that influence the pain sensation instead of drawing further attention to the pain sensation [32]. Pain intensity score is known to be a poor indicator of clinically important pain [33]; therefore, we aim to assess pain impact on daily life experiences and factors that impact pain experiences, which has been reported to be a much more promising approach [34]. Summarizing these data is the simplest way to provide personalized feedback. The advantage of digitalization is the ability to instantly aggregate patient-generated data. Dynamic charts will be built into the app to allow individual relations between pain, emotional, behavioral responses, and coping strategies to be displayed. If the patient wishes, pain data can be shared with their health care provider through a secure portal.

EMMA will be designed to incorporate 8 web-based self-management treatment modules, which is consistent with the design of other face-to-face or web-based self-management programs for chronic pain [35-38]. The lessons are completed over an 8-week period, each week a lesson is unlocked. The 1-week gap between lessons will allow participants time to revisit the content in each lesson, view the resources, and practice the skills. The EMMA intervention will specifically target a number of important aspects of pain self-management relevant for older adults. The EMMA modules will contain: (1) information about realistic goal setting and progress monitoring; (2) chronic pain and self-management strategies; (3) the role of the attention focusing and shifting attention with guided meditation; (4) the role of negative thoughts or beliefs and learning to change them by engaging in pleasant meaningful activities; (5) the role of relaxation and stress management; (6) the role of physical activity, safe ways of getting active, yoga, stretching for older adults, pacing activities, improving well-being through mindfulness, and savoring; (7) learning to regulate emotions, dealing with pain flare-ups and setbacks, psychiatric comorbidities relevant for older adults with chronic pain and (8) and self-management strategies, when to seek help, decreasing social isolation, and incorporating strategies to learn to live with the pain. Patients will receive daily task lists with 3 to 5 small tasks to fulfill in 1 week in order to complete the entire lesson, which will encourage accountability and keep participants motivated. All participants will receive regular email communications to notify them that a new lesson is available.

#### Development Process

In order to maximize usability and acceptability, end-users should be active partners in the design of the product [39]. The development of the platform will follow a user-centered design framework, which includes the user in all phases of system development in an iterative manner. This project will also use a participatory design approach (also called co-design), which allow users to become cocreators of the final system [40]. Indeed, this approach brings all the people involved in the development (eg, researchers and developers), clinicians, and end-users together. EMMA will be built as an embedded web app, appearing to the user as a native app while allowing for compatibility on most mobile devices and rapid updates when necessary. The app will be designed to work on different screen size of devices (responsive web design for phones, tablets, and laptops). The app comprises information shown to the user and input from the users. The web app will be developed using Angular framework that is typescript and HTML-based for the front-end. The back-end it will be developed using Django, a Python-based framework. The connection will be made using an application programming interface that connects the front-end and back-end. The database is based on a MySQL server that is administrated through the Django administration tool.

The project will follow European [22] and Swiss [21] Guidelines for data privacy and security as well as on Responsible Research and Innovation. Privacy by design will be accomplished by putting technical measures in place from the start.

#### Co-development

We will actively involve older adult participants in the developmental process to test the first prototype of the app, after a demonstration of the main features, for 2 consecutive weeks. We will include 10 older adults with chronic pain who use a smartphone or tablet device and meet the following inclusion criteria: (1) age 65 and older; (2) able to read, write, and communicate in French; and (3) able to give written consent. After a testing period for 2 consecutive weeks, a workshop with all study participants will be organized for the co-design process. In order to establish important features of app design for older people, the following topics will be discussed: easy handling of the app, navigation problems, age-appropriate design, auditory support, and other supportive measures. All 3 features (psychoeducational video content, digital pain diary, and self-management modules) will be tested. A meeting (face-to-face or via telephone) will be organized with all consenting participants in order to download and set up the EMMA app on the participant’s personal device, provide participants with app training, and brief them to use the app for 14 days. Participants will be shown all features of the app, until they are comfortable, by research assistants of the University of Fribourg. In addition, participants will be advised to contact one of the investigators if they require assistance when using the app throughout the 14-day period. Participants will be advised to use the app as desired during the testing period but will be asked to use the app at least once daily, perform a daily pain or mood log entry, and go through all psychoeducational content and modules. It is important to note that, during this testing period, there is no requirement of how much participants must use the app. The user feedback will be implemented in the app, and modifications will be discussed in the subsequent group meeting. From previous experience, we expect 2 to 3 rounds will be necessary.

### Feasibility Study

#### Study Purpose and Specific Aims

Feasibility studies are carried out prior to a main study to estimate important parameters required to design the main study, whereas a pilot study is a smaller version of the main study carried out to evaluate if all of the components of the main study can work together [41]. The primary goal of this study is to evaluate the feasibility, usability, and acceptability of the EMMA intervention in a sample of older adults with chronic pain. Because this is a feasibility study, a sample size calculation is not appropriate. However, consistent with other comparable studies [42-44], a target of 30 older adults will be used. Study protocol, intervention, participant information, and informed consent will be reviewed by the Ethics committee of the Canton Vaud, and all participants will provide written informed consent. The study will be conducted according to the principles of the 2013 Declaration of Helsinki.

#### Participants

We plan to include older adults with chronic pain who meet the following inclusion criteria: (1) age 65 years or older; (2) with a chronic pain condition based on International Classification of Disease 11th revision [45] defined as persistent primary pain, persistent posttraumatic or postsurgical pain, persistent neuropathic pain, persistent malignant pain, persistent headache and orofacial pain, persistent visceral pain, and persistent musculoskeletal pain; (3) no signs of neurocognitive disorder (Mini Mental State Examination [46] score >23); (4) able to read and write in French; (5) able to give written informed consent. Exclusion criteria: (1) a current psychiatric disorder (other than depression or anxiety) (2) suicidal thoughts, or (3) a diagnosed neurocognitive disorder. The app's download and use are free for the participants.

#### Procedure and Design

Screening, recruitment, and consenting processes for this study will be carried out by trained research assistants from the University of Fribourg. Prospective participants will be sought from associations catering to older adults such as *Gerontopôle Fribourg* or *Pro Senectute*, using flyers, brochures, and informational talks given at the associations and at the Department of Neuroscience and Movement Science at the General Hospital Fribourg (JC). To facilitate online recruitment, a Facebook page will be created for the entire project. Interested participants will screened to assess eligibility. Eligible participants will be asked to provide written informed consent.

#### Screening and Diagnostic Measures

All participants will undergo the same screening procedure, including a standardized questionnaire to test the general inclusion and exclusion criteria, a structured clinical psychiatric interview for the diagnosis of mental disorder (Structural Clinical Interview for Diagnostic and Statistical Manual for Mental Health [47]), and Mini Mental State Examination [46]. The assessment will be performed by trained research assistants from the University of Fribourg and will be supervised by a senior clinician (KL). We will ask participants to provide sociodemographic data (including age, sex, education, marital status), information on their smoking, drinking, physical activity, self-perception of aging, basic and instrumental activities of daily living, and health care use associated with the pain problems in the past 3 months. Basic and instrumental activities of daily living are each measured with 7 in a modified version of the OARS Multidimensional Functional Assessment Questionnaire [48].

#### Intervention Delivery

All participants will receive individual in-person training on how to use the mobile app in order to minimize possible errors of manipulation. Participants will be advised to use the EMMA app as desired to complete the EMMA intervention (8-week web-based program). Furthermore, participants will be asked to complete the daily diary in the app, assessing pain levels and psychological symptoms including mood, stress, thoughts, behaviors, and activities 3 times per day for 2 weeks before the intervention and 2 weeks after the EMMA intervention in line with other ecological momentary assessment protocols in chronic pain research [49]. Participants will receive an alarm 3 times a day (morning, afternoon and evening) at times determined by the participant, with the requirement that the chosen times be at least 3 hours apart and at least 3 hours after awakening in the morning to allow adequate sampling of variables across the course of the day. Each log will be date- and time-stamped in the app. Studies [50] of participants using electronic devices typically show high rates of adherence at the time of the scheduled prompts; in clinical populations, 85% or more of prompts were answered. We will inform the participants (during the coaching session for the use of the app) about the importance of regularly entering data; participants with less than 50% of data entries will be excluded from analysis. We will continuously monitor the participants on the regularity of their responses to the prompts, and in case of nonadherence, we will contact them and remind them about the importance of entering the data regularly.

#### Outcome Measures

Study measures were chosen based on psychometric properties, including sensitivity to change, brevity, and appropriateness for use with community-dwelling older adults with chronic pain as well as in accordance with recommendations by the Initiative on Methods, Measurement, and Pain Assessment in Clinical Trials group [51]. Outcomes will be measured before and after the intervention with exception of the usability and acceptability measures, which will be measured only after the intervention.

The primary outcome is the Pain Disability Index [52]. The intensity of symptoms associated with pain and depression, including pain, fatigue, and sleep disturbance, will be assessed using the Pain Disability Index [52] to measure the degree to which pain interferes with function in major life areas.

Secondary outcomes will also be measured. The Brief Pain Inventory short form [53] is a validated, widely used, self-administered questionnaire developed to assess the severity of pain and the impact of pain on daily functions. For this study, pain intensity is measured by calculating the mean of 4 items in which respondents are asked to rate their average, current, least, and worst pain during the previous week on a scale from 0 (no pain) to 10 (pain as bad as you can imagine [54]).

The Hospital Anxiety and Depression Scale [55] is a 14-item Likert scale measure; 7 items assess the severity of depression, and 7 items assess the severity of anxiety.

The Geriatric Depression Scale [56] is a 30-item self-report measure specifically designed to assess depressive symptoms in older persons. Good sensitivity and specificity for detecting depression in geriatric psychiatric and medical outpatients has been demonstrated (84%-100% sensitivity, 73%-96% specificity) [57]. The Geriatric Depression Scale was selected because of its screening efficiency with geriatric outpatient populations and its focus on affective rather than physical symptoms.

The World Health Organization Quality of Life [58] 26-item questionnaire measures quality of life in 4 domains, with a 5-point Likert Scale: physical health, psychological health, social relationships and environment

The Pain Coping Strategies Questionnaire [59] is one of the most widely used measures for pain coping and catastrophizing. Measures derived from the Pain Coping Strategies Questionnaire have been shown to be associated with various measures of functioning among patients with different pain conditions [60-62]. The Pain Coping Strategies Questionnaire has demonstrated reliability and validity in several samples of older adults, including those who are older than 75 [63].

#### Usability and Acceptability

After the trial period, participants will complete questionnaires to evaluate the usability and acceptability of the EMMA intervention in healthy older adults, using the Technology Acceptance Model [64] and its version adapted for older adults [65], System Usability Scale [66], and the Emotional Metric Outcomes scale [66]. In addition, all participants will be invited to take part in a semistructured telephone interview at the end of the intervention period. The semistructured interview will focus on evaluating the acceptability of the intervention. Participants will specifically be asked about patterns of app use, including frequency and timing, and their experiences, attitudes, and perspectives on integrating the EMMA intervention into their pain self-management strategies. Participants’ perceptions of the barriers to and facilitators of use of the intervention will be explored. The interviews will be audiorecorded and transcribed verbatim for analysis.

#### Statistical Analysis

We will utilize SPSS software (version 27 or higher; IBM Corp) for management and analysis of data and the latest version of HLM for the Ecological monitoring data (version 8; Scientific Software International Inc). Before analysis, all outcome variables will be tested for normality using the Kolmogorov-Smirnov test and, if necessary, transformed to obtain a normal distribution. Differences in variables (demographic, diagnostic, pain-related) will be compared using *t* tests for continuous variables and chi-square tests for categorical variables. For the primary and secondary outcomes, participants self-reported pain disability, pain severity and impact of pain on daily function, depression, anxiety, quality of life, and pain coping strategies will be compared before and after the intervention. Data from the pain diary yield intensive longitudinal data that are clustered as they represent series of measurements that stem from different individuals. in keeping with current consensus [67] on how to analyze experience sampling data, we will use a multilevel approach to analyze these data, which takes into account clustering and provides flexible tools to investigate within-subject phenomena, such as responses to stressors or pain shifts. We will use software (the latest version of HLM software) that allows for simultaneous within-subject and between-subject modeling, examination of associations among individual difference variables, and examination of individual differences within-subject parameters. In line with similar studies [42], we will use an inductive thematic content analysis for the qualitative interviews. Transcribed interviews will be read and reread to promote immersion in the data and close examination of the interview content. Preliminary themes and subthemes will be generated and continually refined.

## Results

The study is currently ongoing. Development of the digital pain diary has started. Psychotherapeutic web-based app content has been digitalized. Recruitment of participants for the co-design workshops will start as soon as we have a functioning prototype of the electronic pain diary and EMMA intervention, which is to be expected in September 2021. The feasibility study will start as soon as the co-design process is finished and all required changes have been implemented into the pain diary and the EMMA intervention. We expect to start the feasibility study at the beginning of 2022.

## Discussion

### General

This article describes a research protocol for a development and feasibility study of a web-based psychological intervention for older people with chronic pain to support pain self-management. We will develop a self-guided web-based program for chronic pain that consists of an electronic pain diary and a web-based smartphone app with psychotherapeutic treatment modules that will be tested for feasibility as well as providing the first indicators of efficacy. The EMMA intervention combines a strong body of evidence using cognitive behavioral intervention designs with contemporary mHealth principles, allowing for the intervention to be used when and where it matters the most. Potential benefits for older adults with chronic pain are invaluable as this app will address most barriers for effective treatment of older people with chronic pain. This feasibility study should provide information for further optimization of the intervention, and if successful, this app will be tested in an RCT and could become a model to address the needs of older people with chronic pain in Switzerland.

We believe that developing an web-based self-management tool for pain that is suited for older adults will address a major gap for the treatment of pain in older adults. This system could serve as a tailored and customized education tool for individualized services and as a social support and social engagement tool for enhanced pain management. Furthermore, this system could be a potential method of communicating more effectively with health care providers, with the caveat that providers might not have adequate time. Relaying real-time pain status to providers may provide a support tool for patients to convey the daily pain status to their social network and prompt their network for motivation and coping strategies. Furthermore, we also aim to empowering users with this system by supporting them in actively managing their own condition, at their convenience, by self-monitoring to develop a set of self-management strategies.

### Limitations

#### Discomfort With Technology

Although many older adults use mobile technologies, many do not. Moreover, those who do use these technologies often use fewer features than young adults and are easily confused by the wealth of potential apps [68]. Therefore, we will incorporate older adults in the development process to ensure that the app can be navigated easily. We will also provide brief orientation sessions for each participant, where they learn to navigate the app’s features. This process will be continued until the participant is comfortable with their ability to access and navigate the app interface. Most importantly, we aim to minimize these limitations during phase 1 by receiving and iterating on participants feedback related to the app. We will use Web Content Accessibility 2.0 guidelines to ensure to meet the needs of older people (in terms of visibility, design, and navigation). In particular, we will use a large font for text in form fields and other controls to accommodate older adults with visual impairments. Because some older adults lose contrast sensitivity, we will adapt high contrast colors will be used. For the videos, transcripts will be provided to account for eventual hearing loss, background noise, and distractions. Due to possible cognitive decline and inexperience with browsing habits, navigation, and presentation of the program, the organization of pages will be kept as simple as possible. Furthermore, pop-ups, new windows, and tabs will be limited to avoid confusion. Lastly, there will be no time restrictions to complete the transactions.

#### Operational Challenges

Conducting this study will involve several operational challenges. The first challenge is the recruitment of an adequate number of older people with chronic pain who are willing to participate. The second challenge is the nonuse of the intervention. Previous studies have shown that nonadherence is a common problem in web-based interventions [69]. To prevent nonusage, we have taken several measures. Patients will receive reminders when they have not logged into the web-based app. The app includes daily inspirational quotes and blogs to encourage daily use. In addition, log data enable us to track the amount of time patients spend using the intervention. Finally, guidance by the study personnel is offered throughout the intervention to motivate patients, answer questions, and overcome difficulties.

## Abbreviations

mHealth: mobile health
